# Foliar spraying of boron prolongs preservation period of strawberry fruits by altering boron form and boron distribution in cell

**DOI:** 10.3389/fpls.2024.1457694

**Published:** 2024-08-27

**Authors:** Lei Zhang, Changgang Sun, Hui Tian, Jiamin Xu, Xiuwen Wu

**Affiliations:** ^1^ Yantai Academy of Agricultural Sciences, Yantai, China; ^2^ Zhaoyuan Agricultural Technology Promotion Center, Zhaoyuan, China; ^3^ College of Resources and Environmental Sciences, Qingdao Agricultural University, Qingdao, China; ^4^ College of Resources and Environmental Sciences, Shenyang Agricultural University, Shenyang, China

**Keywords:** boron, strawberry, fruit softening, cell wall, subcellular distribution

## Abstract

Boron (B), an essential micronutrient for fruit development, also plays a crucial role in maintaining the shelf life of strawberries (*Fragaria ananassa* Duch.) by affecting cell wall structure and components. We investigated the distribution pattern of B within cells and cell walls in strawberry fruits under different B levels and revealed the relationship between the B distribution in cell walls and fruit firmness after harvesting. Foliar spraying of 0.1% H_3_BO_3_ promoted the growth of strawberry seedlings and improved fruit yield and flesh firmness by 45.7% and 25.6%. During the fruit softening and decay process, the content of bound B and cell wall-B decreased while more B was allocated to the protoplast and apoplast. The changes in B distribution in cells were attributed to cell damage during fruit decay, and B extended the freshness period of the fruits by alleviating the decrease of B distribution in cell walls. After leaving the fruits at room temperature for 10 h, the B content in different cell wall components significantly decreased, while foliar spraying of B alleviated the reduction of B content in covalently bound pectin (CBP), cellulose, and hemicellulose. Meanwhile, B spraying on fruit decreased the activity of cell wall degradation enzymes, including polygalacturonase (PG) and pectin lyase (PL), by 20.2% and 38.1%, while enhancing the demethylation of pectin by increasing pectin methylesterase (PME) activity from 21.6 U/g to 25.7 U/g. Thus, foliar spraying of 0.1% H_3_BO_3_ enhances the cross-linking of B with cell wall components and maintains cell wall structure, thereby prolonging the shelf life of strawberry fruits.

## Highlights

- Foliar spraying of boron improved flesh firmness and prolonged shelf life of strawberry fruits.
- Boron alleviated the decrease of B in CBP, cellulose and hemicellulose in fruits during fruit softening and decay.
- Boron inhibited cell wall degradation by affecting activities of PG, PL, and PME.

## 1 Introduction

Strawberry (*Fragaria ananassa* Duch.) is one of the most widely cultivated and important berries in the world, known for its bright colors, unique flavors, and rich nutrient content (Zhang et al., 2022). It is often referred to as the “fruit queen” due to its significant nutritional and economic value. However, after harvest, strawberries have a short shelf life due to physiological factors such as low fruit firmness and high respiratory intensity. These factors make storage, preservation, transportation, and sales challenging, and they can adversely affect the flavor and nutritional value of the fruits (Chen et al., 2022). As a result, numerous scientists have devoted their attention to understanding the internal mechanisms underlying strawberry fruit ripening and softening, seeking ways to extend the postharvest preservation period of strawberries (Quesada et al., 2009; Li et al., 2020; Shi et al., 2023).

As an essential trace nutrient, boron (B) is crucial not only for the improvement of quality and yield (Aslihan et al., 2011; Singh et al., 2007; Wójcik and Mariusz, 2003), but also plays important roles in the formation and development of reproductive organs in plants (Iwai et al., 2006; Jiang et al., 2007). Numerous studies on apple, pear, tomato, dragon fruit, etc., have shown that application of B was effective in delaying fruit softening and enhancing post-harvest firmness by affecting the activities of cell wall degrading enzymes such as cellulase, pectate lyase (PL), β-galactosidase (β-Gal), polygalacturonase (PG), α-L-arabinofuranosidase (AFase), and pectin methyl esterase (PME) (Kobra et al., 2017; Mohebbi et al., 2020; Gholamnejad et al., 2023). Boron in plants exists in three different forms: water-soluble B, semi-bound B, and bound B (Du et al., 2002). Water soluble B is the main B form in the apoplast, while semi bound B is stored in cells, and bound B is mainly located in pectin polysaccharides in cell walls (Liu et al., 2018). The content and relative distribution of various forms of B in plants are related to plant tissues and external B supply levels. It is widely recognized that B in higher plants primarily binds to cell walls, holding great significance in maintaining the normal structure of cell walls (Matoh et al., 1996; Riaz et al., 2018; Wu et al., 2020). Several studies have demonstrated that B deficiency results in cell wall swelling and an increase in cell wall porosity (Ishii et al., 2001; Kaku et al., 2002). Thus, the distribution of different forms of B in cells and the distribution of B in different cell wall components may induce changes in fruit parenchyma and firmness by affecting fruit cell wall structure and major chemical components (Chen et al., 2011; Lin et al., 2020; Wang et al., 2018; Zhang et al., 2019). Currently, in the process of strawberry cultivation, there is a strong focus on nitrogen, phosphorus, and potassium fertilizers, while medium and trace element fertilizers are often neglected. Therefore, the distribution pattern of B within cells and cell walls in strawberry fruits remains unclear. Additionally, how the distribution pattern of B enhances firmness and prolongs the shelf life of post-harvested strawberry fruits is not yet understood.

This study aims to uncover the effects of foliar B on the different forms of B and the subcellular distribution pattern of B within strawberry fruits. Furthermore, we aimed to explore the crucial role of the B distribution pattern in delaying post-harvest ripening and softening of strawberries. Ultimately, the purpose of the present study was to provide scientific theoretical guidance and innovative ideas for the rational application of B fertilizers in strawberry cultivation.

## 2 Material and methods

### 2.1 Experiment design and materials

This experiment was conducted in the solar greenhouse at Qingdao Agricultural University. The growth medium used was a composite matrix consisting of peat, vermiculite, and perlite mixed in a 2:1:1 ratio. After sterilizing the composite matrix with a 40% solution of Carbendazim (diluted 800 times) and covering it with plastic film for 8 days, the mixture was transferred into plastic pots of 30 cm in height, 20 cm in upper diameter, and 15 cm in bottom diameter). Strawberry seedlings exhibiting uniform growth (with a fresh weight of approximately 20 g and a diameter of 0.5 cm to 0.6 cm) were carefully selected and planted in the plastic pots. After transplantation, each pot was provided with 500 mL of nutrient solution lacking B, and throughout the management stage of the strawberry seedlings, 500 mL of B-free nutrient solution was applied every 10 days. The experiment included two distinct B treatments: control (CK) involving foliar spraying of distilled water and the B treatment involving foliar spraying of a 0.1% H_3_BO_3_ solution. Each treatment was replicated 10 times with 10 strawberry seedlings. Starting from the flowering period, strawberry leaves were treated with distilled water and 0.1% H_3_BO_3_ solutions every 7 days until the strawberries were harvested.

### 2.2 Determination of agronomic traits

After measuring the soil and plant analyzer development (SPAD) value of 10 leaves from each treatment, the seedlings were separated into roots, leaves, and fruits. Initially, we measured the length of the root system using a graduated ruler and weighed the fresh weight of the roots, leaves, and fruits using an electronic balance. Subsequently, the roots, leaves, and a portion of the fruits were dried in an oven at 80°C until a constant weight was achieved. Lastly, the samples were ground, and additional measurements were taken after determining their dry weight using an electronic balance.

### 2.3 Determination of fruit firmness

The fresh strawberry fruits of each B treatment were randomly divided into two portions. The firmness of one portion (10 fruits) was immediately determined using the GY-1 fruit firmness tester (with a probe diameter of 3.5 mm), and the firmness of the other portion (10 fruits) was determined after being left at room temperature for 10 h. Based on the outer edge of the fruit equator, two points of each fruit were measured using the GY-1 fruit firmness tester. Finally, the fruit firmness of each treatment was calculated based on the average value of 20 points.

### 2.4 Extraction of B with different forms from strawberry fruits

According to the method previously reported by Liu et al. (2011), fruits after harvesting 0 h and 10 h were crushed, and immediately shaken and extracted with distilled water for 24 h at 25°C. The filtrate obtained after filtration was the test solution for water-soluble B (WSB). Then the residue was shaken and extracted with 1 mol/L NaCl for 24 h at 25°C, and the filtrate obtained after filtration was the test solution of semi-bound B (SBB) in the cytoplasm. Finally, the residue was shaken and extracted with 1 mol/L HCl for 24 h at 25°C, and the filtrate was the test solution for bound B (BB) in the cell walls.

### 2.5 Extraction of different subcellular fractions of fruits

Strawberry fruits after harvesting 0 h and 10 h were fractionated into cell walls, organelles, and cell soluble fractions using the method outlined by Weigel and Jäger (1980). After fresh fruits were finely ground in a precooled quartz mortar with a homogenate solution of sucrose (0.25 mmol/L), Tris–HCl (50 mmol/L), and dithiothreitol (1 mmol/L), the mixed solutions were centrifuged at 2,000×*g* for 10 min at 4°C and filtered using an 80 μm nylon sieve cloth. The precipitates were cell wall materials. The supernatants were then centrifuged at 12,000×*g* for 45 min at 4°C, and the fragments were organelles, whereas the supernatants were cell soluble fractions.

### 2.6 Extraction of different cell wall components of fruits

Following the approach previously described by Hoson et al. (2003), the cell wall materials were further divided into covalently bound pectin (CBP), ion-bound pectin (IBP), hemicellulose, and cellulose. First, the fully dried and ground cell walls were vibrated and extracted in 0.5 mol/L imidazole solution (pH 7.0) for 24 h at 25°C and centrifuged for 10 min (10,000×*g*). The supernatant was collected, and the residue continued to be vibrated and centrifuged two more times. The supernatant collected three times was defined as CBP. Then precipitates were continuously vibrated in 50 mmol/L Na_2_CO_3_ solution [containing 20 mmol/L trans-1, 2-cyclohexenediaminetetraacetic acid (CDTA)] for 24 h at 25°C and centrifuged for 10 min (10,000×*g*) for three times. The supernatant collected three times after centrifugation was defined as IBP. After adding 4 mol/L KOH solution (containing 0.1% NaBH), the precipitates were continuously extracted at 25°C for 3 h and centrifuged at 10,000×*g* for 10 min. The obtained supernatant was hemicellulose. Finally, the precipitate was washed with 0.03 mol/L acetic acid and alcohol and dried at 60°C to a constant weight, resulting in cellulose.

### 2.7 Determination of B content in plants

The ground samples of organs, subcellular fractions, cell wall components, and solutions of different forms of B were digested in concentrated HNO_3_ for 2 h at 200°C. Subsequently, the B content was determined using inductively coupled plasma mass spectrometry (ICP-MS) (NexIONTM 350X; PerkinElmer, MA, USA) as described by Wu et al. (2021).

### 2.8 Determination of cell wall related enzymes

Fresh fruits were collected and ground to measure the activity of polygalacturonase (PG), β-galactosidase (β-GAL), pectin lyase (PL), and PME using the available commercial PG-1-G, β-GALB-1-Y, PL-1-G and PME-2-G kits (Suzhou Comin Biotechnology Co., Ltd.), respectively.

### 2.9 Data statistics and analysis

The difference between control and B treatments was analyzed using Student’s t-test through SPSS 25.0, and * indicates a significant difference between the two B treatments at the level of *P <*0.05. The significance of differences in data between different B levels and different storage times of fruits was measured using analysis of variance (ANOVA) through SPSS 25.0 software, and lowercase letters indicate significant differences between different treatments at the level of *P <*0.05.

## 3 Results

### 3.1 Strawberry growth and development under different B conditions

Compared to the control treatment, foliar spraying of 0.1% H_3_BO_3_ significantly enhanced the growth and development of strawberry seedlings, resulting in increased root length, SPAD value of leaves, fresh and dry weight of leaves and roots, as well as a higher fruit yield (**Table 1**). By applying B to the leaves, the fruit yield per plant increased by 45.7%.

**Table 1:** The agronomic traits of strawberry plants under different B levels.

Treatment	Root length (cm)	SPAD value	FW of leaf (g/plant)	FW of root (g/plant)	yield of fruit (g/plant)	DW of leaf (g/plant)	DW of root (g/plant)
CK	26.11	29.93	35.51	8.34	71.50	8.33	1.54
B	43.62*	41.8*	52.53*	13.56*	103.62*	14.61*	2.05*

FW represented fresh weight, DW represented dry weight. N = 10. * indicates significant differences (p <0.05) between different B treatments.

### 3.2 The B content in different organs of strawberry

**Figure 1:**
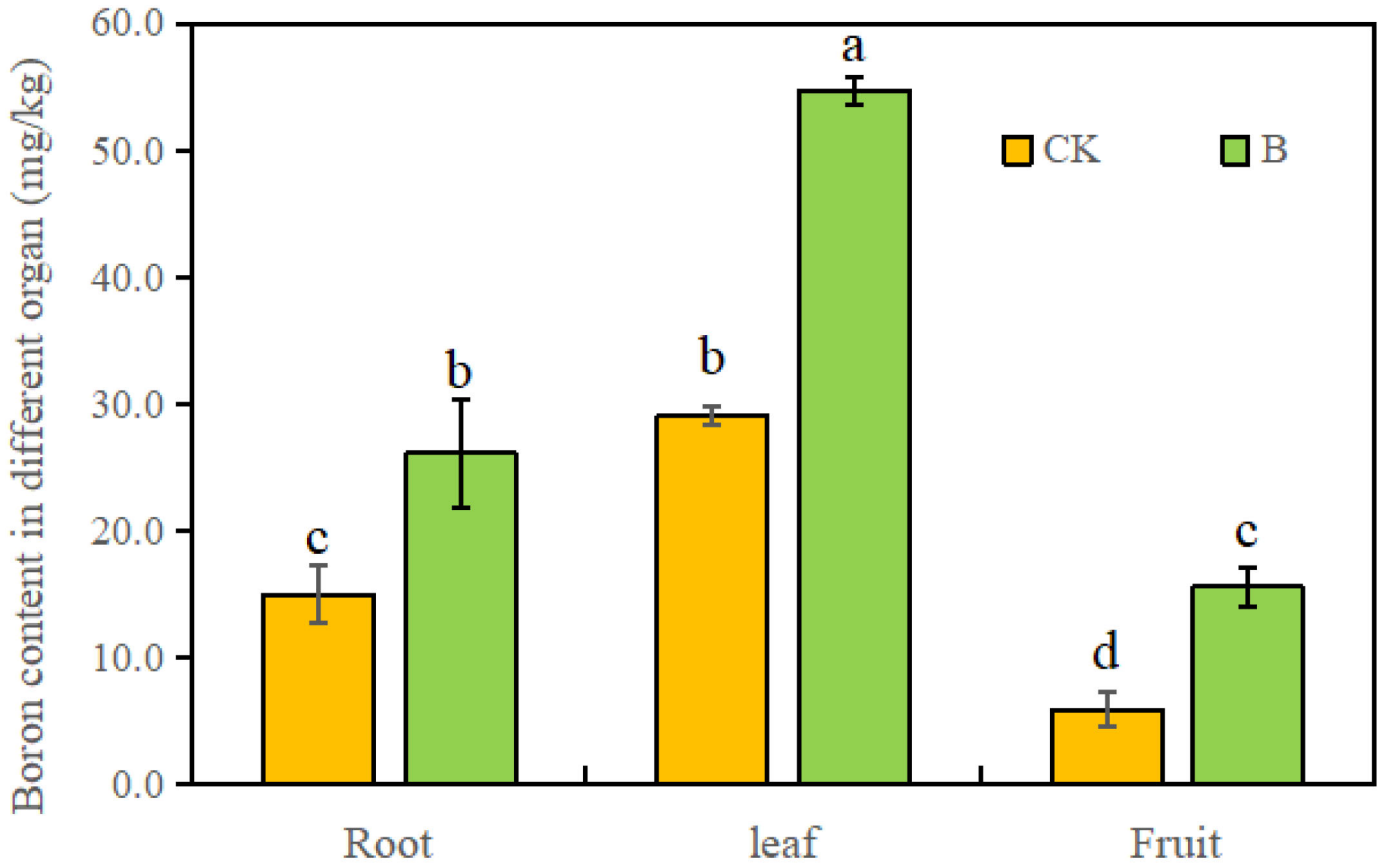
The B content in different organs of strawberry under different B levels. Note: n = 10. The data were presented as mean ± SE, and (a, b, c, d) indicate significant difference (*p <*0.05) between different organs and different B treatments.

In both the control or B treatment, the B content in leaves was the highest. Compared to the control, spraying strawberry leaves with 0.1% H_3_BO_3_ significantly increased the B content in roots, leaves, and fruits by 62.4%, 86.2%, and 128.6%, respectively (**Figure 1**), indicating a higher improvement of B content in strawberry fruits.

### 3.3 Effects of B on strawberry fruit firmness

**Figure 2:**
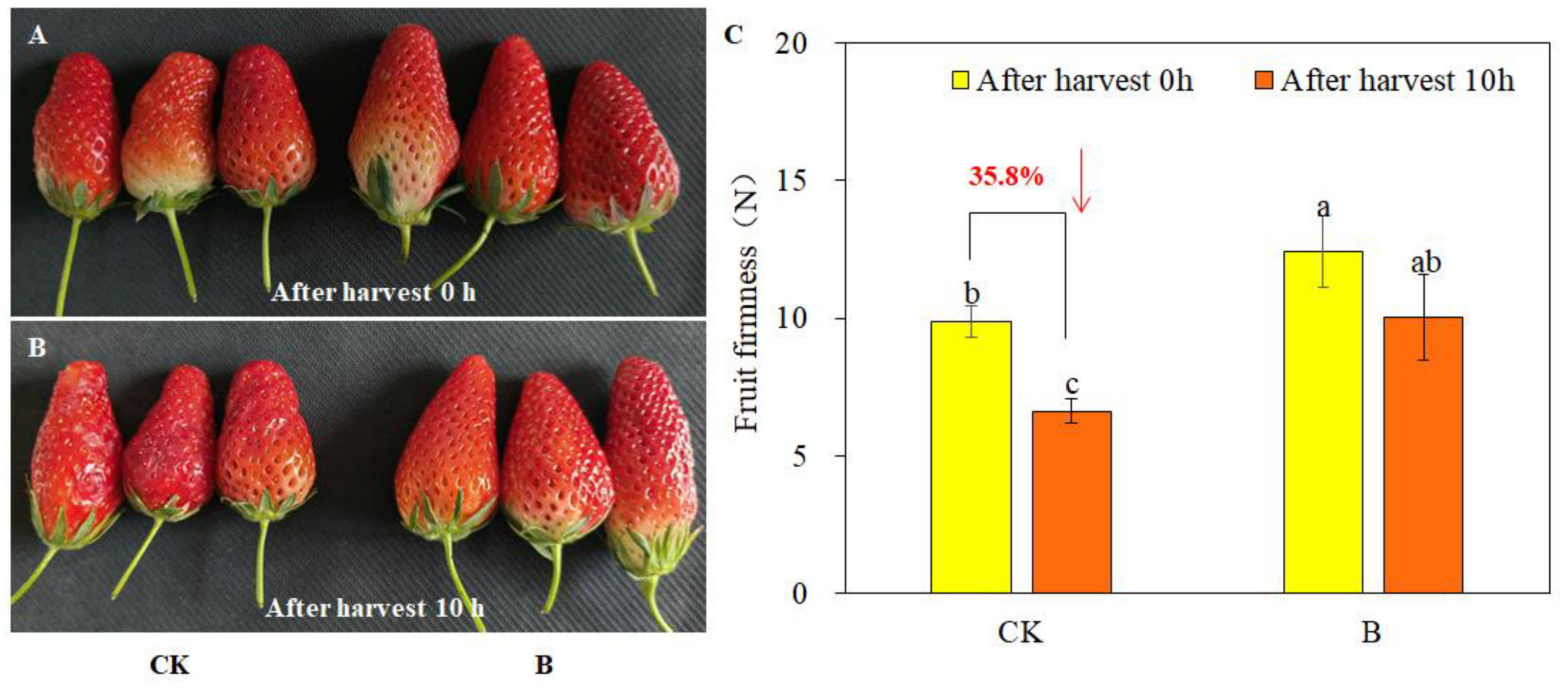
Strawberry fruit phenotype and firmness after harvest 0 h and 10 h under different B levels. **(A)** Just harvested strawberry fruits; **(B)** Strawberry fruits stored at room temperature for 10 h; **(C)** Changes of fruit firmness after storing for 10 h between different B treatment. Note: n = 10. The data were presented as mean ± SE, and (a, b, c) indicate significant differences (*P <*0.05) between different B treatments and harvest time.

The firmness of strawberry fruits was assessed both immediately after harvest and after allowing the fruits to rest at room temperature (22 °C–25 °C) for 10 h. As depicted in **Figure 2A**, after 10 h, the fruits without B spraying showed obvious decay, while the fruits with B spraying still maintained a fresh and healthy state. Furthermore, foliar spraying of B increased the firmness of both of newly harvested fruits and fruits that had been left for 10 h. The calculation results showed that 10 h after the fruits were harvested, the fruit firmness of the control decreased by 35.8%, while the fruit firmness of B treatment only decreased 19.3% (**Figure 2B**). The results indicated that foliar spraying of B may extend the storage time of strawberry fruits.

### 3.4 The content of B in different forms in strawberry fruits

To study the effects of foliar B application on B distribution in fruits, we determined the content of water-soluble B, semi-bounded B, and bounded B. Boron treatment increased the content of B in different forms, especially of bounded B in newly harvested fruits and fruits left for 10 h. However, after leaving the fruits at room temperature for 10 h, the content of bounded B significantly decreased, while the content of water-soluble B and semi bounded B increased (**Table 2**). By calculating the increase/decrease proportion of different forms of B, we found that foliar spraying of B alleviated the change in B distribution in fruits during storage.

**Table 2:** The content of B in different forms in strawberry fruits.

Time	Treatment	WSB content (mg/kg)	SBB content (mg/kg)	BB content (mg/kg)	Change proportion of WSB (%)	Change proportion of SBB (%)	Change proportion of BB (%)
After harvest 0 h	CK	0.20c	0.18d	0.30c	–	–	–
	B	0.37b	0.31b	0.70a	–	–	–
After harvest 10 h	CK	0.31b	0.25c	0.20d	58.23a	34.25a	32.50a
	B	0.48a	0.37a	0.56b	30.61b	18.40b	20.28b

WSB, SBB, and BB represent water-soluble B, semi-bound B, and cell wall bound B, respectively. n = 6. a, b, c, d indicate significant differences (p <0.05) between different harvest times and different B treatments.

### 3.5 The B content and distribution in different subcellular components of fruits

**Figure 3:**
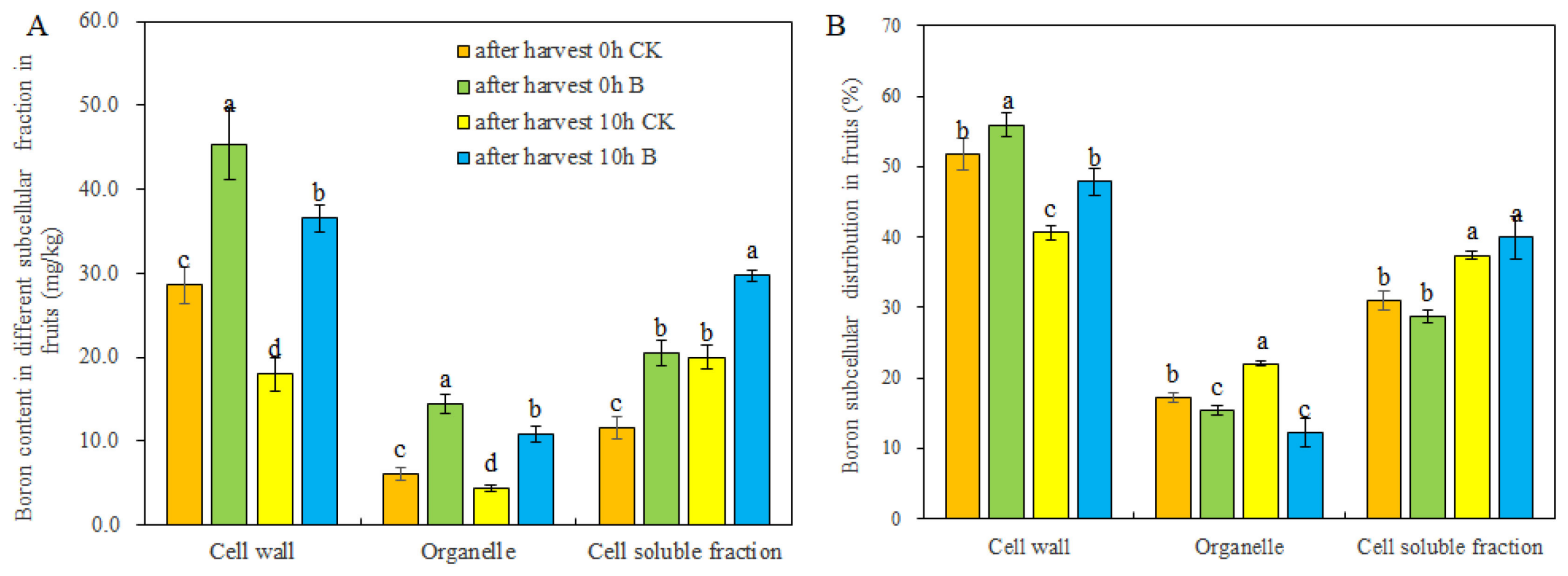
The B content **(A)** and distribution ratio **(B)** in subcellular level of fruits after harvest 0 h and 10 h under different B levels. Note: n = 6. The data were presented as mean ± SE, and (a, b, c, d) indicate significant differences (*p <*0.05) between different harvest times and different B treatments.

The analysis of B content in various subcellular components (cell walls, organelles, and soluble fractions) of the fruits demonstrated a significant increase in B content in all these components upon the application of B through strawberry foliar spraying (**Figure 3A**). After leaving the fruits for 10 h, regardless of control or B treatment, the B content in cell walls and organelles decreased, while the B content in cell soluble fractions increased. However, B treatment reduced the change of B content in different cell fractions of fruits during storage (**Figure 3A**). Notably, the application of B led to a considerable improvement in the B distribution rate within the cell walls and inhibited the distribution of B to the organelles in newly harvested fruits and fruits stored for 10 h (**Figure 3B**). Furthermore, after storage for 10 h at room temperature, the B allocated in cell walls was released to the cell soluble fraction.

### 3.6 The B allocation in different cell wall components in fruits

**Figure 4:**
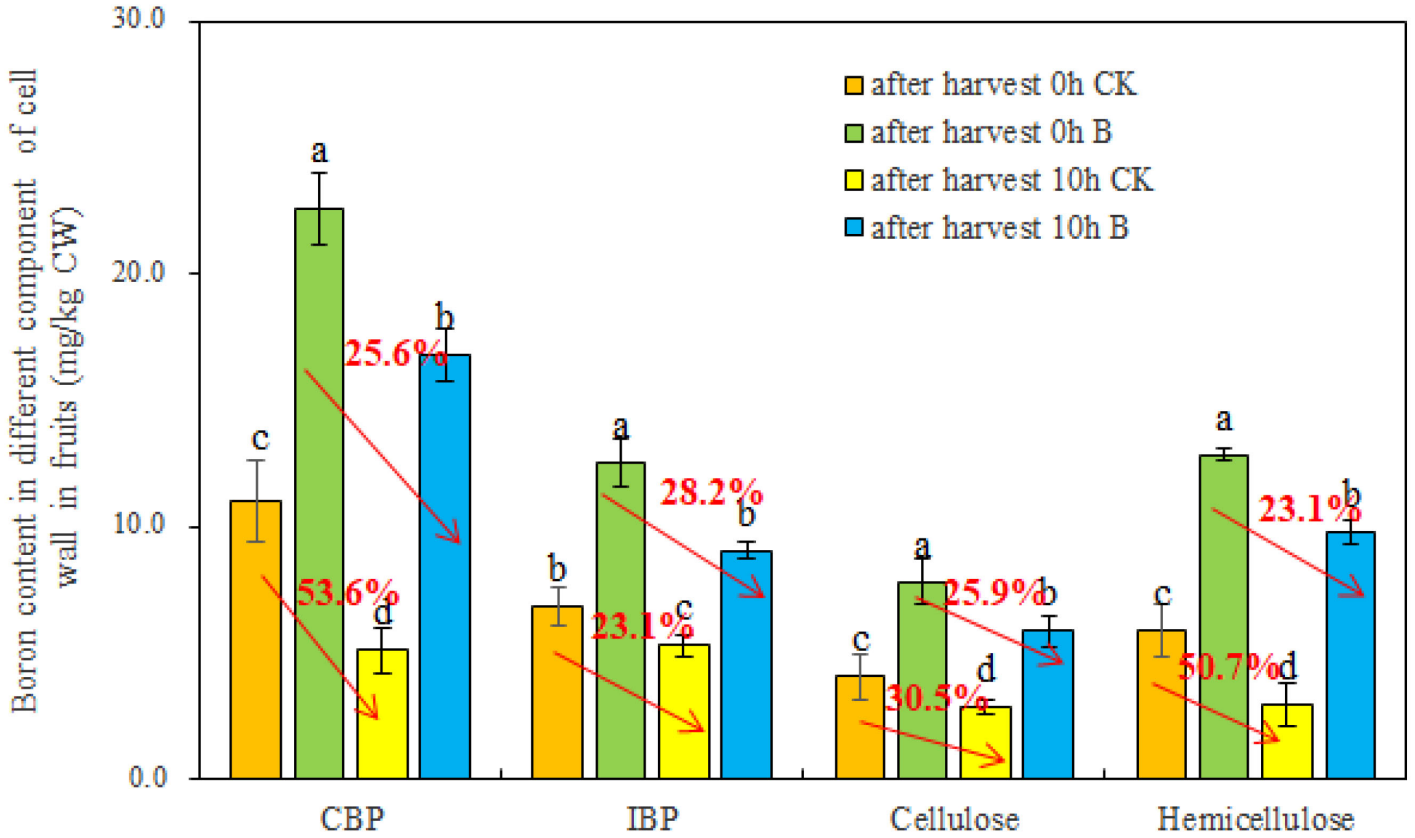
The B content in different cell wall components of strawberry fruits under different B levels. CBP and IBP represented covalently bound pectin and ion-bound pectin, respectively. n = 6. The data were presented as mean ± SE, and (a, b, c, d) indicate significant differences (*p <*0.05) between different harvest time and different B treatments.

To ascertain the distribution of B among different cell wall components in strawberry fruits under varying B levels, the fruit cell walls were segregated into covalently bound pectin (CBP), ion-bound pectin (IBP), cellulose, and hemicellulose. Subsequently, the B content within these cell wall components was quantitatively determined using ICP-MS. As depicted in **Figure 4**, the foliar application of B substantially increased B content in CBP, IBP, cellulose, and hemicellulose. Meanwhile, after 10 h of storage at room temperature, the B content in different cell wall components decreased significantly, regardless of whether the fruits were in the control or B treatment group. Interestingly, compared to the control, foliar spraying of B alleviated the reduction of B content in CBP, cellulose, and hemicellulose in fruits after 10 h.

### 3.7 Effects of B on pectinase activity in strawberry fruits 10 h after harvest

**Figure 5:**
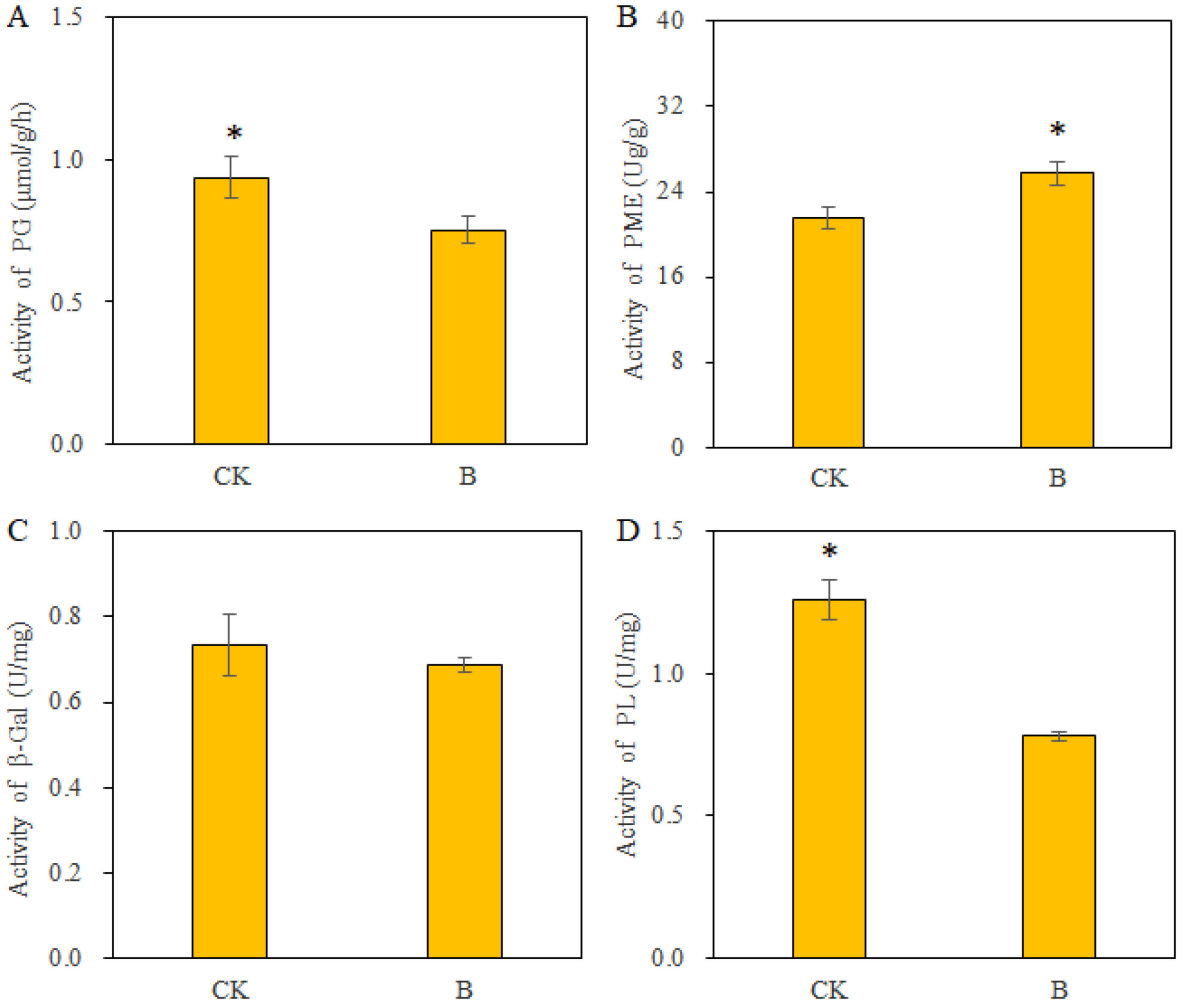
The pectinase activity in strawberry fruits under different B levels. **(A)** polygalacturonase (PG) activity; **(B)** pectin methylesterase (PME) activity; **(C)** β-galactosidase (β-Gal) activity; **(D)** pectin lyase (PL) activity. n = 6. The data were presented as mean ± SE. * indicates significant differences at *P <*0.05 level.

Several cell wall-degrading enzymes, including PG, PL, PME, and β-Gal, contribute to fruit softening by influencing the composition and structure of the cell wall. The results in **Figure 5** indicate that the application of B to strawberries significantly reduced the activity of PG and PL while increasing the activity of PME; however, it had no impact on β-Gal activity. The effects of B on PG and PL were helped mitigate pectin degradation and promote pectin’s demethylation modification. Increased PME can promote pectin demethylation and facilitate the linkage of B with the cell walls and maintenance of a stable cell wall structure, ultimately delaying the softening of strawberry fruits.

## 4 Discussion

Boron (B), an essential nutrient for higher plants, contributes to increasing the yield and quality of fruits by promoting fruit setting, growth, and metabolism (Chouliaras et al., 2009; Abdollahi et al., 2011; Xu et al., 2021; Bons and Sharma, 2023). In this study, foliar spraying of 0.1% H_3_BO_3_ significantly enhanced plant growth and increased fruit fresh weight (**Table 1**). These results were mainly attributed to B’s crucial role in plant fertilization and metabolism (Fang et al., 2019; Michailidis et al., 2023). Research on various crops has found that the primary binding site for B is the cell walls (Chormova et al., 2014; Pan et al., 2012). Our study on strawberry fruits showed that the content of bounded B (BB) in cell walls was higher than that of water-soluble B (WSB) and semi-bounded B (SBB) (**Table 2**). The B content and B allocation in different subcellular fractions (**Figure 3**) also confirmed that more B was distributed to cell walls. In the meantime, B supplication resulted in a higher allocation proportion of cell wall-B in strawberry fruits whether harvested immediately or after 10 h at room temperature (**Figure 3B**), indicating that the distribution of B in different subcellular fractions and cell wall components varies under different external B supply levels (Feng et al., 2022). After 10 h, the content of bounded B and B in cell walls of fruits under different B levels decreased, and more B was released to the apoplasts and soluble fractions. However, the changes in B subcellular distribution were smaller in strawberry with foliar B spraying (**Table 2** and **Figure 3A**). Promoted cross-linking of Ca with cell walls can help maintain normal cell wall structure (Liu et al., 2017; Wigati et al., 2023), which may significantly contribute to extending the storage time of fruits. It has been reported that differences of B allocation influence the cell wall structure and composition of plants (Liu et al., 2018; Wu et al., 2017). Thus, the binding of B to cell wall components may be also closely related to fruit softening (**Figure 2**). The degree of fruit ripening and softening is primarily assessed by fruit firmness. Foliar application of B to strawberries increased fruit firmness at initial harvest and notably delayed the decline in firmness after 10 h (**Figure 1**), which may contribute to the preservation and storage of strawberry fruits (Jiang et al., 2019).

Boron can bind to different cell wall components (pectin, cellulose, and hemicellulose), with pectin being the primary binding site for B in the cell walls (Yang et al., 2005; Liu et al., 2018). Our results showed that the B content in CBP was the highest in fruits across all treatments (**Figure 4**). The B allocation in the cell walls of strawberry fruits varied under different external B supply levels and was affected by storage time (**Figure 3**). However, it is unknown how foliar spraying of B influences the B content in different cell wall components in strawberry fruits and how it affects fruit softening. For strawberries, B spraying during plant growth stages promoted the cross-linking of B with different forms of pectin (IBP, CBP), cellulose, and hemicellulose (**Figure 4**). However, after 10 h at room temperature, the B content in different cell wall polysaccharides of fruits significantly decreased (**Figure 4**), suggesting cell wall degradation, disruption of reticular polymers, alterations in cell wall structure (Du et al., 2018; Wu et al., 2017), reduced cell tension, and ultimately, fruit softening (Bakshi and Ananthanarayan, 2020). Interestingly, compared to the control, B spraying slightly reduced the B content in IBP, CBP, cellulose, and hemicellulose of strawberry fruits after 10 h at room temperature (**Figure 4**). This indicates that foliar application of B to strawberries alleviated cell wall degradation and contributed to the preservation and storage of strawberry fruits (Mohebbi et al., 2020; Gholamnejad et al., 2023).

A variety of pectinases, including polygalacturonase (PG), β-galactosidase (β-Gal), pectin lyase (PL), and pectin methylesterase (PME), collectively impact the cross-linking structure among cell wall components (Saffer, 2018; Bajpai, 2022). The depolymerization caused by pectinases involved in cell wall degradation leads to the breakdown of cell wall components, disruption of reticular polymers, alterations in cell wall structure, reduced cell tension, and ultimately, fruit softening (Bakshi and Ananthanarayan, 2020). Investigations into PG gene expression across various fruits, such as strawberries (Shi et al., 2023), peaches (Ming et al., 2016), tomatoes (Yang et al., 2022), and figs (Wang et al., 2023), have substantiated the link between fruit softening and PG activity. Additionally, PG and PME work together within cell walls to facilitate fruit softening, as highly methylated pectin needs PME demethylation before being hydrolyzed by PG (Shi et al., 2023). However, the dynamics of PME activity during the softening process differ across various fruit types and varieties. For instance, PME activity progressively increases during the ripening and softening of bananas, while it decreases in papayas (Ali et al., 2004). The significant role of PL is to participate in the cleavage of pectin structure during fruit softening and ripening (Marín‐Rodríguez et al., 2002). The PL gene is regarded as a potential candidate for enhancing fruit hardness and impeding softening through molecular approaches (Jiménez-Bermúdez et al., 2002). Although numerous studies have explored inhibiting pectinase activity using exogenous substances to slow down fruit softening and flavor changes, such as procyanidin (Chen et al., 2022), 1-methylcyclopropene (Win et al., 2021), and calcium (Liu et al., 2017; Huang et al., 2023), early-season B spraying on apples did not affect the activity of PG, PME, PL, or β-Gal, but resulted in slight changes in fruit flesh firmness (Mohebbi et al., 2020). A study on tomatoes indicated that B application increased fruit firmness while decreasing pectin methylesterase activity (Gholamnejad et al., 2023). Previous studies have proposed that B promotes pectin synthesis and demethylation while inhibiting pectin degradation in rapeseed roots by suppressing the activity of pectinase enzymes (PG, PL, PME, and β-Gal) (Wu et al., 2020). For strawberries, B spraying during plant growth stages promoted the binding of cell wall-bound B in fruits of newly harvested and those left at room temperature for 10 h. This may be the result of the diminished activity of pectinases involved in pectin degradation (PG, PL) and promoted pectin demethylation (PME) in fruits (**Figure 5**).

## 5 Conclusions

The foliar application of 0.1% H_3_BO_3_ to strawberries effectively enhanced plant growth, fruit yield, and quality. Furthermore, B extended the shelf life of strawberry fruits by alleviating cell wall degradation, attributed to facilitated B cross-linking with pectin, cellulose, and hemicellulose, thereby aiding in the maintenance of cell wall structural integrity. These findings offer promising theoretical insights and innovative techniques for retarding the softening and deterioration of strawberry fruits while prolonging their post-harvest preservation period through the strategic use of B application.

## Data Availability

The raw data supporting the conclusions of this article will be made available by the authors, without undue reservation.
